# Using routinely recorded data in a UK RCT: a comparison to standard prospective data collection methods

**DOI:** 10.1186/s13063-021-05294-6

**Published:** 2021-07-05

**Authors:** G. A. Powell, L. J. Bonnett, C. T. Smith, D. A. Hughes, P. R. Williamson, A. G. Marson

**Affiliations:** 1 Department of Molecular and Clinical Pharmacology, Clinical Sciences Centre, Lower Lane, Fazakerley, Liverpool, L9 7LJ UK; 2 Department of Biostatistics, University of Liverpool, Waterhouse Building, Block F, 1-5 Brownlow Street, Liverpool, L69 3GL UK; 3 Liverpool Health Partners, Liverpool, L69 3GL UK; 4 Centre for Health Economics & Medicines Evaluation, Bangor University, Ardudwy, Normal Site, Gwynedd, North Wales LL57 2PZ UK

**Keywords:** Routine data, Administrative data, Agreement, Randomised controlled trial

## Abstract

**Background:**

Routinely recorded data held in electronic health records can be used to inform the conduct of randomised controlled trials (RCTs). However, limitations with access and accuracy have been identified. *Objective*: Using epilepsy as an exemplar condition, we assessed the attributes and agreement of routinely recorded data compared to data collected using case report forms in a UK RCT assessing antiepileptic drug treatments for individuals newly diagnosed with epilepsy.

**Methods:**

The case study RCT is the Standard and New Antiepileptic Drugs II (SANAD II) trial, a pragmatic, UK multicentre RCT assessing the clinical and cost-effectiveness of antiepileptic drugs as treatments for epilepsy. Ninety-eight of 470 eligible participants provided consent for access to routinely recorded secondary care data that were retrieved from NHS Digital Hospital Episode Statistics (*N*=71) and primary and secondary care data from The Secure Anonymised Information Linkage Databank (*N*=27). We assessed data items relevant to the identification of individuals eligible for inclusion in SANAD II, baseline and follow-up visits. The attributes of routinely recorded data were assessed including the degree of missing data. The agreement between routinely recorded data and data collected on case report forms in SANAD II was assessed using calculation of Cohen’s kappa for categorical data and construction of Bland-Altman plots for continuous data.

**Results:**

There was a significant degree of missing data in the routine record for 15 of the 20 variables assessed, including all clinical variables. Agreement was poor for the majority of comparisons, including the assessments of seizure occurrence and adverse events. For example, only 23/62 (37%) participants had a date of first-ever seizure identified in routine datasets. Agreement was satisfactory for the date of prescription of antiepileptic drugs and episodes of healthcare resource use.

**Conclusions:**

There are currently significant limitations preventing the use of routinely recorded data for participant identification and assessment of clinical outcomes in epilepsy, and potentially other chronic conditions. Further research is urgently required to assess the attributes, agreement, additional benefits, cost-effectiveness and ‘optimal mix’ of routinely recorded data compared to data collected using standard methods such as case report forms at clinic visits for people with epilepsy.

**Trial registration:**

Standard and New Antiepileptic Drugs II (SANAD II (EudraCT No: 2012-001884-64, registered 05/09/2012; ISRCTN Number: ISRCTN30294119, registered 03/07/2012))

**Supplementary Information:**

The online version contains supplementary material available at 10.1186/s13063-021-05294-6.

## Background

There is great expectation that the analysis of routinely recorded healthcare data will provide rapid and efficient answers to healthcare questions and be a vehicle to generate health and wealth for the UK, exemplified by the recent UKRI investment in Health Data Research UK [1] and collaboration between Novartis and National Health Service England to conduct a novel large scale clinical trial using healthcare system data [2]. It is vital therefore that we understand their utility in clinical trials in common chronic diseases, epilepsy being the exemplar in this paper.

Routinely recorded data can be defined as data that are routinely recorded for specific, defined primary purposes, other than audit or research [3]. Data regarding clinical care are routinely documented in electronic medical records and stored in administrative healthcare databases in the UK [4, 5].

Routinely recorded data have established use in retrospective observational studies such as record linkage population studies, but their use in randomised controlled trials (RCTs) is less well established. RCTs remain the gold standard for assessing the efficacy and effectiveness of treatments in healthcare [6] and publicly funded, pragmatic RCTs typically provide longer-term outcome data to inform chronic disease management. However, the majority of RCTs are time-consuming and resource-intensive as clinicians typically assess participants at clinic visits and record trial data on case report forms. If a trial is assessing outcomes that are important to participants, such as a core outcome set [7, 8], one might expect relevant data to be recorded routinely.

Routinely recorded data have been used to inform judgements about the feasibility, sample size and recruitment targets in RCTs [9], measure certain participant outcomes [10–12] such as mortality and inform health economic analyses [13]. Routinely recorded data are a potential source of data for a wider range of clinical outcomes, and their use could greatly improve the efficiency of clinical research [5, 10, 14], reducing the burden to participants and researchers [15]. Furthermore, data from non-clinical routine sources may inform outcomes beyond the standard RCT assessments of clinical efficacy and effectiveness. For example, cost data (such as the use of healthcare resources) and socio-economic data (such as employment and means-tested benefits data) may inform health economic analyses and the assessment of the broader societal impact of healthcare interventions.

Limitations in feasibility, accessibility and accuracy have been identified [16, 17]. For example, the accuracy of routinely recorded data in identifying incident cases may be reduced compared to prevalent cases which may impact on the utility of routinely recorded data to identify individuals with ‘new’ diagnoses, a frequent target group for RCTs. Furthermore, routinely recorded data may have limitations in identifying recurrent events compared to single events. For example, it may be expected that the identification of stroke would be of greater accuracy than the identification of seizure occurrences.

The accuracy of diagnosis of epilepsy using routinely recorded healthcare data compared to an independent review of patients’ medical records has been assessed [18]. However, there is scant evidence of the assessment of accuracy or agreement compared to standard methods of data collection employed in prospective research, such as the record of data on Case Report Forms (CRFs).

Routinely recorded data are being used increasingly in prospective research, including RCTs, without evidence of an appraisal for this purpose [19]. An assessment of the attributes and agreement of routinely recorded data compared to data collected using standard prospective methods is therefore urgently required.

### Objective

To assess the attributes and agreement between routinely recorded data and data collected using case report forms in a UK pragmatic RCT assessing antiepileptic drug treatments for individuals newly diagnosed with epilepsy.

## Methods

The case study RCT is the Standard and New Antiepileptic Drugs II (SANAD II (EudraCT No: 2012-001884-64, ISRCTN Number: 30294119)) trial, a pragmatic, UK multicentre RCT assessing the clinical and cost-effectiveness of selected antiepileptic drugs as first-line treatments for newly diagnosed epilepsy.

Individuals with newly diagnosed epilepsy participating in SANAD II, aged 16 years or older and with a minimum of 12 months follow-up, were eligible for inclusion in this study. Participants were sent a study invitation via post and asked to sign a consent form. One further postal invitation was sent if there was no initial response. Routinely recorded data were retrieved from NHS Digital [20], which included data for episodes of patient contact with NHS secondary care in England and from The Secure Anonymised Information Linkage Databank (SAIL) [21], including access to data for episodes of patient contact with NHS secondary care and in selected cases primary care for patients in Wales. All datasets used clinical coding systems, the inpatient and outpatient datasets using the International Statistical Classification of Diseases and Related Health Problems (ICD) 10 [22], the primary care dataset using UK READ Codes [23] and the emergency datasets using unique coding systems. The study was reviewed and approved by the North of Scotland Research Ethics Service and Health Research Authority (29/01/16, REC reference: 16/NS/0007, Protocol number: UOL001183, IRAS project ID: 189002).

To permit assessment of the attributes of routinely recorded data and agreement compared to data collected using case report forms, data variables relevant to each of the following aspects were identified or constructed from the available datasets:

- *The identification of individuals meeting the inclusion criteria and eligible for recruitment*
  - ◦ Variables included ‘Age’, ‘Date of First-Ever Seizure’ and ‘Date of Diagnosis of Epilepsy’
- *The collection of data relevant to the baseline RCT assessment*
  - ◦ Variables included ‘Classification of Seizures’ and ‘Clinical Investigation Results’
- *The collection of data relevant to the follow-up RCT assessments*
  - ◦ Variables included ‘Date of First Follow-Up Seizure’, ‘Adverse Events’ and ‘Planned and Unplanned Healthcare Attendances’ together with the constructed outcomes ‘Time to First Follow-Up Seizure’ and ‘Time to 12-Month Remission’

**Fig. 1:**
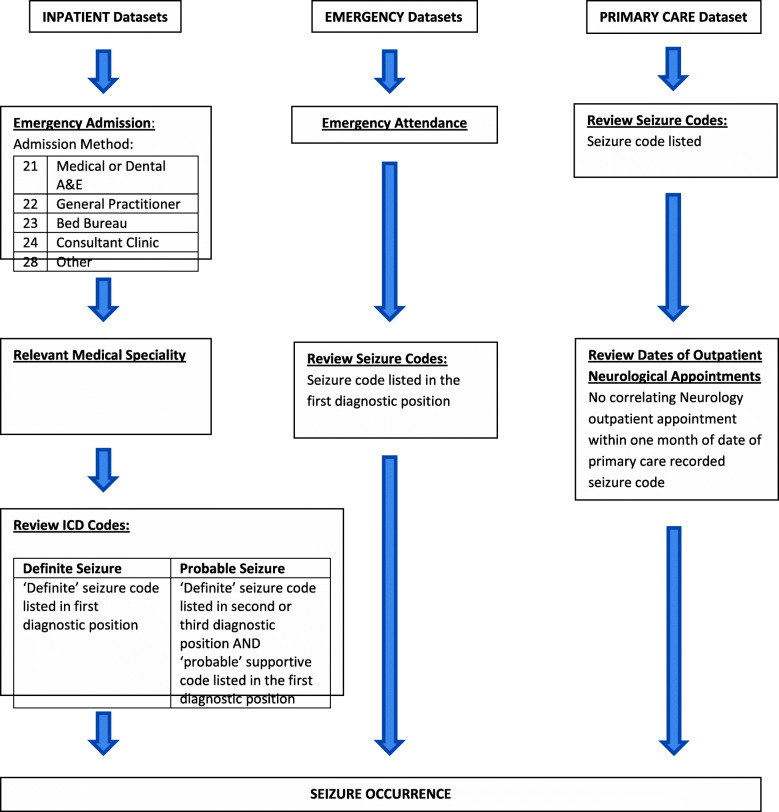
Algorithm for the identification of seizure occurrence

An algorithmic approach was developed for each variable, using knowledge of the coding systems, clinical behaviours and organisational pathways. Similar approaches utilising the clinical interpretation of routinely recorded data have previously been used in studies assessing seizures [24] and in other disease areas in the UK [25–27]. Diagnostic codes indicating the occurrence of relevant events were specified a priori and the ‘best-case’ dataset was used in the analysis, constructed using the available data from all relevant primary and secondary care routine datasets. Throughout the analyses, the included participants were analysed as a complete cohort, without reference to antiepileptic drug (AED) prescribed or SANAD II study treatment arm. The algorithms developed for each variable together with the diagnostic codes are presented as Supplementary Figures 1-4 and Supplementary Tables 1-6. Figure 1 presents as an example the algorithm for the identification of seizure occurrence.

Participants study data recorded using standard methods in SANAD II were retrieved. Data were captured on CRFs at baseline and regular follow-up intervals (3, 6, 9 and 12 months thereafter).

Assessment of the attributes of routinely recorded data included identifying the degree of missing data compared to data collected using case report forms.

A statistical assessment of the agreement was completed. Bland-Altman methods [28] were used to assess the agreement between paired continuous data and acceptable clinical limits of agreement were specified a priori, informed by clinical discussion. The *Difference* and *Mean* between datasets were computed. Bland Altman Plots were constructed with the *Difference* variable plotted on the *Y* axis and the *Mean* variable plotted on the *X* axis. The mean of the *Difference* variable was plotted and the 95% confidence limits of agreement were calculated and discussed in the context of the specified acceptable clinical limits of agreement [28]. Time to event outcomes were assessed using Kaplan-Meier curves and a log-rank test was performed with *P* value < 0.05 indicating a statistically significant difference. To assess agreement between paired, nominal categorical datasets and cross tabulations were presented followed by calculation of Cohen’s kappa and a *P* < 0.05 indicated the level of agreement defined by kappa being significant [29]. All analyses were performed in SPSS, version 22.

The STROBE Checklist is the most relevant, available research reporting checklist and has been referred to when drafting this manuscript, the summary flowchart included as an additional file.

## Results

Four hundred and seventy participants in SANAD II were aged 16 years or older with a minimum of 12 months follow-up, fulfilling the inclusion criteria (April 2016). Ninety-eight participants provided consent to participate in this study, 55 males and 43 females with a mean age of 50. Demographics were similar for the 372 patients not providing consent. Routinely recorded data were requested for 71 participants resident in England, with data available in inpatient, outpatient, emergency and critical care datasets and 27 participants resident in Wales, with data available in inpatient, outpatient and emergency datasets. Primary care data were available for a subset of 23 participants resident in Wales.

Table 1 summarises the attributes of the available data in primary and secondary care sources for selected variables in the 23 participants in whom data from both sources were available. As demonstrated, secondary care sources provide more complete data for identifying first seizure occurrence, whilst primary care sources provide more complete data regarding the diagnosis of epilepsy and, in addition, prescribing information. For the analyses presented in this paper, the ‘best case’ dataset has been used including available data from all sources.

**Table 1:** Summary of primary and secondary care data*

Variable	Total participants	Total data in secondary care datasets	Total exclusively in secondary care datasets	Total data in primary care datasets	Total exclusively in primary care datasets
First-ever seizure (all types)	12 (52%)	10 (43%)	6 (26%)	6 (26%)	2 (8%)
Diagnosis of epilepsy (baseline)	18 (78%)	5** (22%)	5 (22%)	13*** (57%)	13 (57%)
Date of first follow-up seizure	7 (30%)	3 (13%)	3 (13%)	4 (17%)	4 (17%)
Date of AED first prescription	23 (100%)	0****	0	23 (100%)	23 (100%)

*For 23 participants in whom data from both primary and secondary care sources were available

**Diagnosis made by record of two seizure episodes

***Diagnosis made by record of a code consistent with ‘diagnosis of epilepsy’

****Prescribing data only available in primary care datasets

The results for each variable and outcome measure assessed are summarised in Table 2. Flowcharts summarising the identification of relevant data, Bland-Altman plots and Kaplan-Meier survival curves are presented in Supplementary Figures 6-34 and Supplementary Tables 7-14.

**Table 2:** Summary of results: quality and agreement

**Variable**	**Total eligible**	**SANAD II total**	**Routine total**	Difference*	Agreement**	Acceptable agreement***
**Data variables relevant to the identification of eligible individuals and baseline assessment in SANAD II**						
First-ever seizure (all Types)	62	62 (100%)	23 (37.1%)	*P*=0.002	BA: − 84.09 (− 313.12–144.94)	30 days
First-ever seizure (tonic-clonic)	62	43 (69.4%)	22 (35.5%)	*P*=0.043	BA: − 28.07 (− 123.72–67.58)	30 days
Diagnosis of epilepsy (baseline)	78	78 (100%)	37 (47.4%)	*P*=0.004	BA: 30.54 (− 106.17–167.25)	30 days
Diagnosis of epilepsy (all-time)	81	81 (100%)	47 (58.0%)	*P*=0.195	BA: − 26.23 (− 294.10–241.64)	30 days
Classification of seizures (baseline)	37	37 (100%)	37 (100%)	N/A	CK: 0.151 (P=0.018)	N/A
Classification of seizures (all-time)	47	47 (100%)	47 (100%)	N/A	CK: 0.123 (P=0.019)	N/A
Clinical investigations Magnetic resonance imaging	98	72 (73.5%)	9 (9.2%)	N/A	CK: 0.016 (P=0.602)	N/A
Clinical investigations Computed tomography	98	33 (33.7%)	27 (27.6%)	N/A	CK: 0.406 (P< 0.001)	N/A
Clinical investigations Electroencephalography	23	18 (78.2%)	8 (34.8%)	N/A	CK: 0.188 (P=0.131)	N/A
**Data variables relevant to the follow-up in SANAD II**						
Date of first follow-up seizure	98	61 (62.2%)	22 (22.4%)	*P*=0.024	BA: − 86.26 (− 386.41–213.89)	30 Days
Date of first follow-up tonic-clonic seizure	98	35 (35.7%)	20 (20.4%)	*P*=0.374	BA: − 9.20 (− 436.46–418.06)	30 Days
Date 12-month remission achieved	98	46 (46.9%)	74 (75.5%)	*P*=0.004	BA: 34.24 (− 115.48–183.96)	30 Days
Date of AED first prescription	26	26	25	*P*< 0.001	BA: − 19.76 (− 67.86–28.34)	90 Days
Adverse events	97	97	2	-	-	-
Planned healthcare attendances Baseline assessment	98	98 (100%)	87 (88.8%)	*P*< 0.001	BA: 1.67 (− 7.21–10.55)	N/A
Planned healthcare attendances Follow-up assessments	350	350 (100%)	317 (90.6%)	*P*< 0.14	BA: 0.07 (− 4.33–4.47)	N/A
Unplanned attendances: emergency	94	52 (55.3%)	37 (39.4%)	*P*=0.051	BA: 0.05 (− 0.734–0.834)	N/A
Unplanned attendances: inpatient	94	12 (12.8%)	19 (20.2%)	*P*=0.098	BA: − 0.02 (− 0.72–0.68)	N/A
**Outcomes relevant to the follow-up in SANAD II**						
**Variable**	**Total eligible**	**SANAD II total**	**Routine total**	**SANAD II mean (95% CI)**	**Routine mean** **(95% CI)**	Difference****
Days to first follow-up seizure	98	61 (62.2%)	22 (22.4%)	325 (258–393)	751 (680–822)	*P*< 0.001
Days to 12-month remission	98	46 (46.9%)	74 (75.5%)	567 (515–618)	393 (375–410)	*P*< 0.001

*Paired *T* test (normally distributed data), Wilcoxon signed-rank (non-normally distributed data)

**Bland-Altman methods (BA) (continuous data) = mean (lower 95% confidence limit of agreement–upper 95% confidence limit of agreement)

**Cohen kappa (CK) (categorical data) = 0.01–0.20 as none to slight agreement, 0.21–0.40 as fair, 0.41– 0.60 as moderate, 0.61–0.80 as substantial and 0.81–1.00 as almost perfect agreement

***Acceptable clinical limit of agreement specified a priori

****Log-rank test

### Variables relevant to the identification of eligible individuals and SANAD II baseline assessment

Sixty-two of the 98 included participants had a date (day, month and year) of first-ever seizure occurrence recorded in SANAD II during the time period covered by the available routine data and were eligible for the assessment of ‘date of first-ever seizure’. In the routine datasets, a first-ever seizure occurrence was identified in 23 of the 62 participants. The most common recorded codes were non-specific ‘seizure’ and ‘epilepsy’ codes. The most common ICD 10 code was ‘Unspecified Convulsions (R568)’, emergency code ‘CNS Conditions – Epilepsy (HES 41, SAIL 17A)’ and READ codes ‘Convulsion NOS (R003z)’ and ‘Had a Fit (IB63)’.

**Fig. 2:**
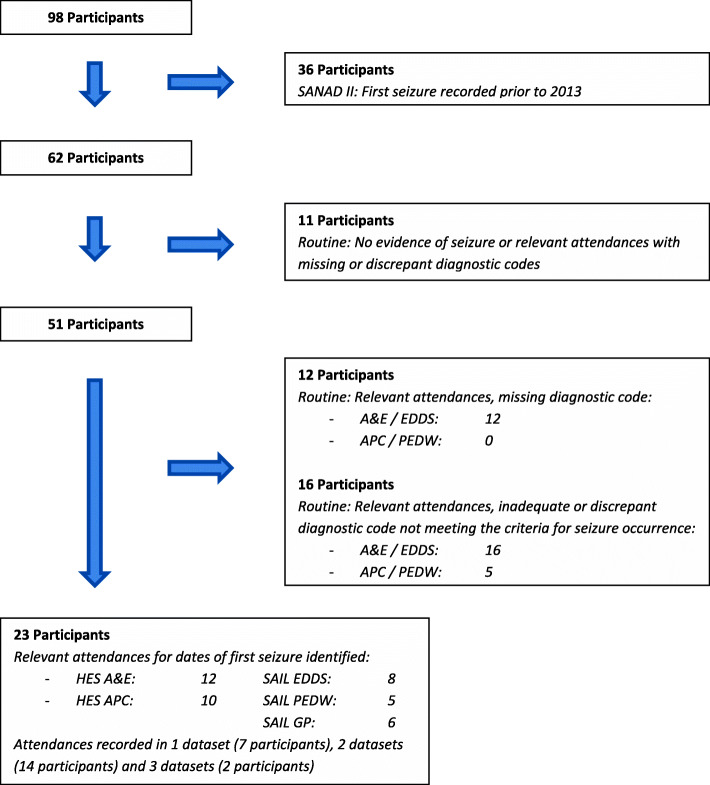
The identification of the date of first-ever seizure in routine datasets

**Fig. 3:**
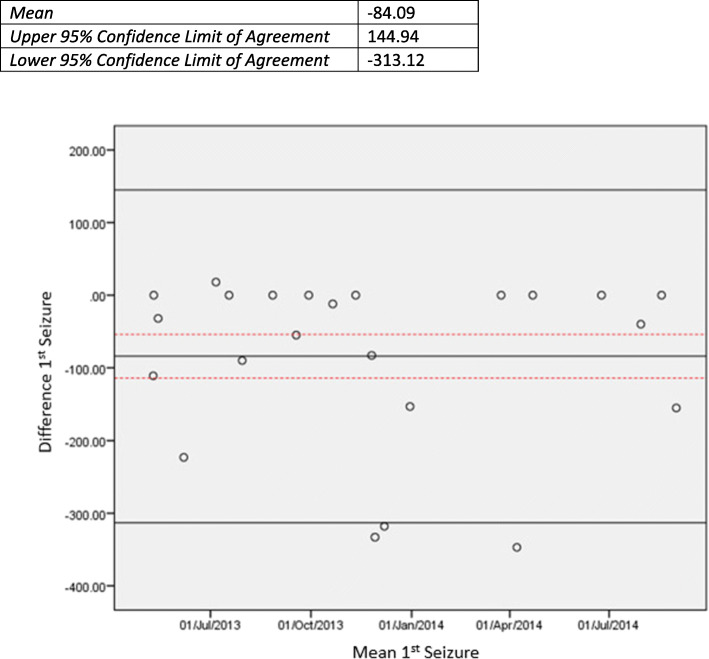
Date of first-ever seizure: Bland-Altman plot

Figure 2 shows a flowchart for the identification of ‘first-ever seizures’ in routine datasets. Although sixteen participants had a relevant attendance within 48 h of a seizure occurrence recorded in SANAD II, seizure could not be identified as the cause of the attendance due to inadequate or discrepant diagnostic codes. Codes included ‘CNS, Non-Epilepsy’ in the emergency datasets and ‘Disorientation’ and ‘Confusion’ in the inpatient datasets. The Bland-Altman plot (Fig. 3) demonstrates that when a date of first seizure is identified in the routinely collected data; agreement with data collected using CRFs is poor. The 95% confidence limits of agreement between the dates of first-ever seizure are 145 and 313 days, well in excess of the specified 30 day clinically acceptable limit. The mean of the difference between the dates is 84, indicating that on average the date of first-ever seizure is identified in the routinely collected data 84 days after the seizure is identified in the SANAD II dataset. Limiting the first-ever seizures to ‘tonic-clonic’ seizures, the data were marginally more complete with seizures identified for 22 out of 43 participants with a tonic-clonic seizure recorded in the SANAD II dataset, although agreement regarding the date of first tonic-clonic seizure occurrence remains poor.

At the time of recruitment into SANAD II, using routinely collected data 41 of 78 participants met the criteria for diagnosis of epilepsy and agreement was poor for the ‘date of diagnosis of epilepsy’. Seizures could be classified in all participants using data retrieved from routinely recorded datasets; however, agreement was poor (Cohen’s kappa 0.151, *P*=0.018) explained by the disproportionately large number of participants deemed ‘unclassified’ as a result of lack of clinical detail in the codes recorded.

### Variables and outcomes relevant to the follow-up in SANAD II

**Fig. 4:**
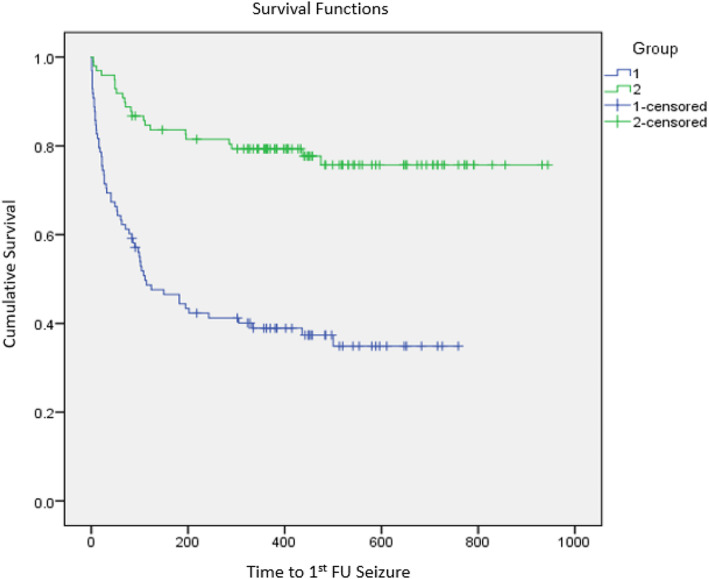
Kaplan-Meier curve: the time to first follow-up seizure

Twenty-two participants had first follow-up seizures identified using routinely collected datasets, compared to 61 participants using SANAD II data. The mean time to first follow-up seizure was 325 days calculated using SANAD II data and 778 days calculated using routine data. Figure 4 presents the Kaplan-Meier curve. Proportionally, a greater number of first follow-up tonic-clonic seizures were identified, 20 participants using routinely collected data compared to 35 using SANAD II.

Data regarding adverse events were sparse in routine datasets, and of 97 adverse events recorded in SANAD II only two were identifiable in routine datasets.

Prescribing data were only available from the primary care dataset. Twenty-six ‘first AED prescriptions’ were identified, all prescription times being within the 90-day clinical limit of agreement with data recorded in SANAD II.

Dates of episodes of outpatient planned and inpatient and emergency unplanned healthcare resource use had fewer missing data compared to clinical variables; for example, 317 outpatient attendances were included in the SANAD II dataset, compared to 350 recorded in routinely collected data. Furthermore, the dates of attendance were within the acceptable clinical limits of agreement.

For some variables, additional data were recorded in the routinely recorded datasets that were not available in the SANAD II CRFs. For three participants in whom the CRF data indicated they were seizure-free, additional seizures were identified in the routine datasets. For one individual without an EEG result on the SANAD II CRF, the routine data indicated that they had an EEG. Two participants of the 23 with available prescribing data in routine datasets were prescribed additional AEDs not recorded in the CRFs. Finally, data regarding AED adherence could be inferred from the routine datasets using the frequency of repeat prescription, noting the assumptions made in reaching this result.

## Conclusions

Routinely recorded data are increasingly being used in clinical trials to provide answers to important clinical questions. However, this study shows that for epilepsy, and potentially therefore for other chronic conditions, it is not currently possible to identify important clinical events and outcomes in routinely recorded data in the UK. Therefore, their exclusive use is not a valid substitute for data collected using standard methods such as case report forms completed at clinic visits or via telephone. There is an ongoing drive to incorporate routinely recorded data into RCTs in an effort to improve research efficiency and reduce the burden for participants [1, 2], and the results of this study, using epilepsy as the exemplar, raise potentially significant concerns about the suitability of routine data for this purpose.

We assessed the use of routinely recorded data to identify individuals eligible for recruitment into a RCT for people with newly diagnosed epilepsy and to collect baseline, follow-up and outcome data.

Regarding seizure occurrence, it was not possible to identify baseline (pre-epilepsy diagnosis) seizures in the routinely collected data for 63% of patients or seizures during follow-up for 64%. When baseline seizures or follow-up seizures were identified, there was a poor agreement with dates recorded in the trial database. The date of the first follow-up seizure identified was 86 days (mean) after the first follow-up seizure recorded in the trial database. Follow-up seizures could not be identified in the routine data, either because no event was documented at all, or because codes used did not indicate that seizure was the reason for attendance or admission. As a consequence, analysis of routinely recorded data grossly underestimates the outcomes ‘time to first seizure’ and ‘time to 12-month remission’.

Similarly, a ‘diagnosis of epilepsy’ was identified in less than half of the participants using the routinely recorded data around the time of randomisation into SANAD II. It was also not possible to classify seizure types for the majority of participants due to inadequate coding and coding options.

Specific codes labelling ‘adverse events’ were not recorded. Furthermore, healthcare attendances correlating with the dates of adverse events recorded in SANAD II were not identified. SANAD II participants self-reported more unplanned emergency attendances and fewer unplanned inpatient admissions, compared to those events identified in routinely recorded datasets. For these data, it may be more likely that the routinely recorded data are correct, and that recall bias is responsible for the discrepancy in the SANAD II dataset.

Explanations for the results may include the inaccurate recording of codes in routinely recorded datasets or inaccurate initial clinical diagnosis of seizures and epilepsy. Furthermore, certain events may not have been ‘recordable’, for example, if participants did not seek medical attention following seizure occurrence or if relevant codes or detail are not included in the available coding systems and routine datasets. Finally, patients with seizures may frequently be treated and then discharged from the emergency department, where diagnostic coding is not mandatory. This study report refers specifically to outcomes as applied to epilepsy, although it must be noted that in other disease areas, similar results may not be found and these explanations may not apply. For example, disease areas where the diagnosis is more explicit, where the presentation is acute and where admission (rather than discharge from an emergency department) is more commonly required may be more likely to be more completely and accurately recorded in routine datasets. Examples may include ‘myocardial infarction’ or ‘stroke’.

The results of this study have implications for the use of routinely recorded data in RCTs in patients with epilepsy. In SANAD II, exclusively using routinely recorded data would not have allowed reliable identification of eligible individuals for recruitment, collection of baseline data or the collection of data for seizure and adverse outcomes [14, 30]*.* Furthermore, only 98 of 470 patients (21%) provided consent for their routine data to be retrieved. Notably, the 470 patients had already consented to participate in the SANAD II RCT.

Whilst routinely recorded data could not be used alone to construct clinical outcomes, routine data could aid the identification of events, such as seizure occurrence or prescription changes, that had been missed, and these data remain important for assessing health service resource use for economic analyses [31]. However, the limitations in accessing these data, such as cost and lengthy application processes, would need to be considered [17].

Further research is urgently required to assess the attributes, additional benefits, feasibility and cost-effectiveness of accessing routinely recorded data during RCTs in the UK in epilepsy and other common disease areas. The ‘Studies Within A Trial’ (SWAT) approach, embedding methodological research within an existing prospective trial [32], could be suggested as the method to facilitate such research. For example, routinely recorded data could be requested for a subset of participants within existing RCTs. The analysis of such data, compared to the standard RCT methods, may directly inform the use of routine data in the ongoing RCT as well as inform future research.

The ‘de-identified’ nature of primary care data, cost and poor geographical coverage were notable limitations. Development of the infrastructure to record national primary care data coverage is required, either through improved collaboration between existing routine data sources with individual-level data linkage or the development of national data sources, such as the NHS Digital General Practice Extraction Service [33]. Further, a number of countries have integrated healthcare systems allowing for national administrative healthcare databases, such as the Swedish Hospital Discharge Register, the Danish National Hospital Register and the Canadian Chronic Disease Surveillance System. In these examples, it is possible to retrieve routinely recorded data from electronic medical records for individuals across hospital inpatient admissions and emergency care, outpatient clinic and primary care attendances. In the UK, standardisation of the coding systems used in different care settings and between datasets and greater involvement of clinicians in the clinical coding process may improve the accuracy and completeness of clinical coding. A suggested proposed improvement could include the development of a national, integrated electronic health record for use in direct clinical care, with secondary uses in audit and research. Such a proposal would include direct clinician input and selection of diagnostic codes and would have significant potential for the improved recording of data and improved utility for the datasets in clinical practice and research.

Whilst patients and the public are broadly supportive of data recording and sharing for healthcare research, concerns remain over confidentiality and potential abuses of data [34]. Public concerns regarding the sharing and linking of routinely recorded data will hamper future efforts to develop linked routinely recorded administrative databases, despite the likely benefits to individuals and the population. Further research is required with public engagement to define the issues of importance to members of the public and assess perspectives with regards to the routine recording of data and subsequent use for secondary purposes including research.

This study has notable limitations. The variables and constructed outcomes derived from the routinely recorded datasets were defined and extracted using algorithms developed for each comparison. There is a risk that relevant clinical events may not have been identified. To address this limitation and explore the data further, the routinely recorded data for each participant were examined in their entirety to identify additional relevant events. This process was feasible as a result of the small sample size. Furthermore, this study involved the retrospective identification of events in routinely recorded datasets and comparison to events identified in a study designed and completed using standard prospective methods. It must be acknowledged that the comparator study was not designed to be completed retrospectively using routinely recorded datasets, and if this had been the case, alternative methodologies may have been employed.

Substantial further development is now required to improve the utility of routinely recorded data in research. To improve the likelihood of significant progress, initiatives for development should include collaboration from the government, National Health Service, researchers and perhaps most importantly acknowledging recent controversies, patients and the public; re-gaining their trust will be essential in realising the individual and population healthcare benefits of routinely recorded data. Examples of such work in progress include the European Health Data and Evidence Network [35], whose objectives include standardising real-world health data Europe-wide for purposes including research, Health Data Research UK [36] and the PED4PED initiative, a project aiming to improve outcomes for patients with epilepsy by linking data between primary, secondary and the emergency services [37].

## Supplementary Information

**Additional file 1.**

**Additional file 2.**

## Data Availability

The study dataset (consisting of SANAD II RCT data and routinely recorded data provided by NHS Digital and SAIL) cannot be shared. This is a result of the risks to individual confidentiality and data security arrangements detailed in the relevant Data Sharing Agreements.

## Abbreviations

AED: Antiepileptic drug
CNS: Central nervous system
CRF: Case report form
HES: Hospital Episode Statistics
NHS: National Health Service
RCT: Randomised controlled trial
SAIL: Secure Anonymised Information Linkage Databank
SANAD II: The Standard and New Antiepileptic Drug Trial II
