# Supplementary material for: Using routinely recorded data in a UK RCT: a comparison to standard prospective data collection methods
Source: Trials. 2021 Jul 5;22:429. doi: 10.1186/s13063-021-05294-6 (PMC8259387; doi:10.1186/s13063-021-05294-6)

*Supplementary Figure 1: Algorithm for the Identification of Seizure Occurrence*

**EMERGENCY Datasets**

**PRIMARY CARE Dataset**

**INPATIENT Datasets**

*Supplementary Table 1: Emergency and READ Codes Included in the Definition of Seizure Occurrence*

**Review Dates of Outpatient Neurological Appointments**

No correlating Neurology outpatient appointment within one month of date of primary care recorded seizure code

**Review Seizure Codes:**

Seizure code listed in the first diagnostic position

**Review Seizure Codes:**

Seizure code listed

**Emergency Attendance**

**SEIZURE OCCURRENCE**

**Review ICD Codes:**

| **Definite Seizure** | **Probable Seizure** |
| --- | --- |
| ‘Definite’ seizure code listed in first diagnostic position | ‘Definite’ seizure code listed in second or third diagnostic position AND ‘probable’ supportive code listed in the first diagnostic position |

**Relevant Medical Speciality**

**Emergency Admission**:

Admission Method:

| 21 | Medical or Dental A&E |
| --- | --- |
| 22 | General Practitioner |
| 23 | Bed Bureau |
| 24 | Consultant Clinic |
| 28 | Other |

| **Code** | **Code Description** |
| --- | --- |
| ***Emergency Codes*** | |
| HES: 241 | CNS Conditions - Epilepsy |
| HES: 24 | CNS Disorder* |
| SAIL: 17A | Seizure / Convulsion |
| SAIL: 17Z | CNS Condition – Unspecified* |
| ***Primary Care READ Codes*** | |
| ***‘Seizure’ Codes*** | |
| 1B1W.00 | Transient epileptic amnesia |
| 1B27.00 | Seizures in response to acute event |
| 1B64.00 | Had a convulsion |
| 1B64.11 | Convulsion - symptom |
| 282..00 | O/E - fit/convulsion |
| 282..11 | O/E - a convulsion |
| 282..13 | O/E - a seizure |
| 2828 | Absence seizure |
| 282Z.00 | O/E - fit/convulsion NOS |
| 667D.00 | Epilepsy control poor |
| 667T.00 | Daily seizures |
| 667V.00 | Many seizures a day |
| 667W.00 | Emergency epilepsy treatment since last appointment |
| F132z12 | Myoclonic seizure |
| F250011 | Epileptic absences |
| F250200 | Epileptic seizures - atonic |
| F250300 | Epileptic seizures - akinetic |
| F251200 | Epileptic seizures - clonic |
| F251300 | Epileptic seizures - myoclonic |
| F251400 | Epileptic seizures - tonic |
| F251600 | Grand mal seizure |
| F253.11 | Status epilepticus |
| F254400 | Epileptic automatism |
| F254500 | Complex partial epileptic seizure |
| F255600 | Simple partial epileptic seizure |
| F25H.00 | Generalised seizure |
| F25X.00 | Status epilepticus, unspecified |
| F25y300 | Complex partial status epilepticus |
| F25z.11 | Fit (in known epileptic) NOS |
| Fyu5200 | [X]Other status epilepticus |
| Fyu5900 | [X]Status epilepticus, unspecified |
| R003.00 | [D]Convulsions |
| R003400 | [D]Nocturnal seizure |
| R003y00 | [D]Other specified convulsion |
| R003z00 | [D]Convulsion NOS |
| R003z11 | [D]Seizure NOS |
| Ryu7100 | [X]Other and unspecified convulsions |
| 1B63.00 | Had a fit |
| 1B63.11 | Fit - had one, symptom |
| 282..12 | O/E - a fit |
| 2822 | O/E - grand mal fit |
| 2823 | O/E - petit mal fit |
| 2824 | O/E - focal (Jacksonian) fit |
| 2824.11 | O/E - Jacksonian fit |
| 2824.12 | O/E - focal fit |
| 2825 | O/E - psychomotor fit |
| R003200 | [D]Fit |
| F252.00 | Petit mal status |
| F253.00 | Grand mal status |
| ***‘Epilepsy’ Codes*** | |
| 1O30.00 | Epilepsy confirmed |
| 667B.00 | Nocturnal epilepsy |
| F035200 | Rasmussen syndrome |
| F132100 | Progressive myoclonic epilepsy |
| F132111 | Unverricht - Lundborg disease |
| F25..00 | Epilepsy |
| F250.00 | Generalised non-convulsive epilepsy |
| F250000 | Petit mal (minor) epilepsy |
| F250100 | Pykno-epilepsy |
| F250400 | Juvenile absence epilepsy |
| F250y00 | Other specified generalised non-convulsive epilepsy |
| F250z00 | Generalised non-convulsive epilepsy NOS |
| F251.00 | Generalised convulsive epilepsy |
| F251000 | Grand mal (major) epilepsy |
| F251011 | Tonic-clonic epilepsy |
| F251500 | Tonic-clonic epilepsy |
| F251y00 | Other specified generalised convulsive epilepsy |
| F251z00 | Generalised convulsive epilepsy NOS |
| F254.00 | Partial epilepsy with impairment of consciousness |
| F254000 | Temporal lobe epilepsy |
| F254100 | Psychomotor epilepsy |
| F254200 | Psychosensory epilepsy |
| F254300 | Limbic system epilepsy |
| F254z00 | Partial epilepsy with impairment of consciousness NOS |
| F255.00 | Partial epilepsy without impairment of consciousness |
| F255000 | Jacksonian, focal or motor epilepsy |
| F255011 | Focal epilepsy |
| F255012 | Motor epilepsy |
| F255100 | Sensory induced epilepsy |
| F255200 | Somatosensory epilepsy |
| F255300 | Visceral reflex epilepsy |
| F255311 | Partial epilepsy with autonomic symptoms |
| F255400 | Visual reflex epilepsy |
| F255500 | Unilateral epilepsy |
| F255y00 | Partial epilepsy without impairment of consciousness OS |
| F255z00 | Partial epilepsy without impairment of consciousness NOS |
| F25A.00 | Juvenile myoclonic epilepsy |
| F25B.00 | Alcohol-induced epilepsy |
| F25C.00 | Drug-induced epilepsy |
| F25D.00 | Menstrual epilepsy |
| F25E.00 | Stress-induced epilepsy |
| F25F.00 | Photosensitive epilepsy |
| F25y.00 | Other forms of epilepsy |
| F25y000 | Cursive (running) epilepsy |
| F25y100 | Gelastic epilepsy |
| F25yz00 | Other forms of epilepsy NOS |
| F25z.00 | Epilepsy NOS |
| Fyu5000 | [X]Other generalized epilepsy and epileptic syndromes |
| Fyu5100 | [X]Other epilepsy |
| SC20000 | Traumatic epilepsy |

***Legend: *:*** *Codes excluded from the assessment of seizures prior to participant diagnosis of epilepsy*

*Supplementary Table 2: ICD Codes Included in the Definitions of ‘Definite’ and ‘Probable’ Seizure Occurrence*

| **ICD 10 Code** | **ICD Code Description** | **Definite** | **Probable** |
| --- | --- | --- | --- |
| G400 | LOCAL-RELATED (PART) IDIOPATH EPILEP/EPILEP SYND WITH SEIZURE | 1 | 0 |
| G401 | LOCAL-RELATED (PART) SYMPTOM EPILEPSY/EPILEPTIC SYND WITH SEIZURE | 1 | 0 |
| G402 | LOCAL-RELATED (PART) SYMPTOM EPILEPSY/ EPILEP SYND | 1 | 0 |
| G403 | GENERALIZED IDIOPATHIC EPILEPSY AND EPILEPTIC SYNDROMES | 1 | 0 |
| G404 | OTHER GENERALIZED EPILEPSY AND EPILEPTIC SYNDROMES | 1 | 0 |
| G405 | SPECIAL EPILEPTIC SYNDROMES | 1 | 0 |
| G406 | GRAND MAL SEIZURES, UNSPECIFIED (WITH OR WITHOUT PETIT MAL) | 1 | 0 |
| G407 | PETIT MAL, UNSPECIFIED, WITHOUT GRAND MAL SEIZURES | 1 | 0 |
| G408 | OTHER EPILEPSY | 1 | 0 |
| G409 | EPILEPSY, UNSPECIFIED | 1 | 0 |
| G410 | GRAND MAL STATUS EPILEPTICUS | 1 | 0 |
| G411 | PETIT MAL STATUS EPILEPTICUS | 1 | 0 |
| G412 | COMPLEX PARTIAL STATUS EPILEPTICUS | 1 | 0 |
| G418 | OTHER STATUS EPILEPTICUS | 1 | 0 |
| G419 | STATUS EPILEPTICUS, UNSPECIFIED | 1 | 0 |
| R568 | OTHER AND UNSPECIFIED CONVULSIONS | 1 | 0 |
| F019 | VASCULAR DEMENTIA, UNSPECIFIED | 0 | 1 |
| F100 | MENTAL AND BEHAVIOURAL DISORDERS DUE TO ACUTE INTOXICATION | 0 | 1 |
| F101 | MENTAL AND BEHAVIOURAL DISORDERS DUE TO HARMFUL USE OF ALCOHOL | 0 | 1 |
| F102 | MENTAL AND BEHAVIOURAL DISORDERS DUE TO ALCOHOL DEPENDENCE | 0 | 1 |
| F103 | MENTAL AND BEHAVIOURAL DISORDERS DUE TO WITHDRAWAL OF ALCOHOL | 0 | 1 |
| F104 | MENTAL AND BEHAVIOURAL DISORDERS AND DELIRIUM | 0 | 1 |
| F419 | ANXIETY DISORDER, UNSPECIFIED | 0 | 1 |
| G439 | MIGRAINE, UNSPECIFIED | 0 | 1 |
| H538 | OTHER VISUAL DISTURBANCES | 0 | 1 |
| J690 | PNEUMONITIS DUE TO FOOD AND VOMIT | 0 | 1 |
| K292 | ALCOHOLIC GASTRITIS | 0 | 1 |
| R402 | COMA, UNSPECIFIED | 0 | 1 |
| R410 | DISORIENTATION, UNSPECIFIED | 0 | 1 |
| R418 | OTHER & UNSPEC SYMPTOMS & SIGNS INVOLVING COGNITIVE FUNCTION | 0 | 1 |
| R42X | DIZZINESS AND GIDDINESS | 0 | 1 |
| R451 | RESTLESSNESS AND AGITATION | 0 | 1 |
| R51X | HEADACHE | 0 | 1 |
| R55X | SYNCOPE AND COLLAPSE | 0 | 1 |
| R600 | LOCALIZED OEDEMA | 0 | 1 |
| R798 | OTHER SPECIFIED ABNORMAL FINDINGS OF BLOOD CHEMISTRY | 0 | 1 |
| S000 | SUPERFICIAL INJURY OF SCALP | 0 | 1 |
| S001 | CONTUSION OF EYELID AND PERIOCULAR AREA | 0 | 1 |
| S008 | SUPERFICIAL INJURY OF OTHER PARTS OF HEAD | 0 | 1 |
| S009 | SUPERFICIAL INJURY OF HEAD, PART UNSPECIFIED | 0 | 1 |
| S010 | OPEN WOUND OF SCALP | 0 | 1 |
| S018 | OPEN WOUND OF OTHER PARTS OF HEAD | 0 | 1 |
| S019 | OPEN WOUND OF HEAD, PART UNSPECIFIED | 0 | 1 |
| S099 | UNSPECIFIED INJURY OF HEAD | 0 | 1 |
| S308 | OTHER SUPERFICIAL INJURIES OF ABDOMEN, LOWER BACK AND PELVIS | 0 | 1 |
| Z038 | OBSERVATION FOR OTHER SUSPECTED DISEASES AND CONDITIONS | 0 | 1 |
| Z739 | PROBLEM RELATED TO LIFE-MANAGEMENT DIFFICULTY, UNSPECIFIED | 0 | 1 |

*Supplementary Figure 2: Algorithm for the Diagnosis of Epilepsy*

**PRIMARY CARE Dataset**

**OUTPATIENT Datasets**

**INPATIENT Datasets**

**EMERGENCY Datasets**

**Review READ Codes:**

Two Seizure codes (any dataset) >24 hours apart

AND

**Review Dates of Outpatient Neurological Appointments**

No correlating Neurology outpatient appointment within one month of date of primary care recorded seizure code

**Review Seizure Codes:**

Two seizure codes (any dataset) in the first diagnostic position >24 hours apart

**Emergency Attendance**

**Review ICD Codes:**

Two Seizure codes (any dataset) >24 hours apart

| **Definite Seizure** | **Probable Seizure** |
| --- | --- |
| ‘Definite’ seizure code listed in first diagnostic position | ‘Definite’ seizure code listed in second or third diagnostic position AND ‘probable’ supportive code listed in the first diagnostic position |

**Review READ Codes:**

Epilepsy code listed

OR

**Review ICD Codes:**

Epilepsy code listed

**Emergency Admission**:

Admission Method:

| 21 | Medical or Dental A&E |
| --- | --- |
| 22 | General Practitioner |
| 23 | Bed Bureau |
| 24 | Consultant Clinic |
| 28 | Other |

**DIAGNOSIS OF EPILEPSY**

**Relevant Medical Speciality**

OR

**Admission**:

Any Admission Method

Any Speciality

**Review ICD Codes**:

Epilepsy code listed in any diagnostic position

*Supplementary Table 3: ICD Codes Included in the Definition of Diagnosis of Epilepsy*

| **ICD 10 Code** | **ICD Code Description** |
| --- | --- |
| **Focal Epilepsy** | |
| G400 | LOCAL-RELATED (PART) IDIOPATH EPILEP/EPILEP SYND WITH SEIZURE |
| G401 | LOCAL-RELATED (PART) SYMPTOM EPILEPSY/EPILEPTIC SYND WITH SEIZURE |
| G402 | LOCAL-RELATED (PART) SYMPTOM EPILEPSY/ EPILEP SYND |
| **Generalised Epilepsy** | |
| G403 | GENERALIZED IDIOPATHIC EPILEPSY AND EPILEPTIC SYNDROMES |
| G404 | OTHER GENERALIZED EPILEPSY AND EPILEPTIC SYNDROMES |
| **Unclassified Epilepsy** | |
| G40 | EPILEPSY AND RECURRENT SEIZURES |
| G405 | SPECIAL EPILEPTIC SYNDROMES |
| G408 | OTHER EPILEPSY |
| G409 | EPILEPSY, UNSPECIFIED |

*Supplementary Table 4: READ Codes Included in the Definition of Diagnosis of Epilepsy*

| **Code** | **Code Description** |
| --- | --- |
| ***Focal Epilepsy*** | |
| F035200 | Rasmussen syndrome |
| F254.00 | Partial epilepsy with impairment of consciousness |
| F254000 | Temporal lobe epilepsy |
| F254100 | Psychomotor epilepsy |
| F254200 | Psychosensory epilepsy |
| F254300 | Limbic system epilepsy |
| F254z00 | Partial epilepsy with impairment of consciousness NOS |
| F255.00 | Partial epilepsy without impairment of consciousness |
| F255000 | Jacksonian, focal or motor epilepsy |
| F255011 | Focal epilepsy |
| F255200 | Somatosensory epilepsy |
| F255311 | Partial epilepsy with autonomic symptoms |
| F255500 | Unilateral epilepsy |
| F255y00 | Partial epilepsy without impairment of consciousness OS |
| F255z00 | Partial epilepsy without impairment of consciousness NOS |
| F25y100 | Gelastic epilepsy |
| F25y000 | Cursive (running) epilepsy |
| SC20000 | Traumatic epilepsy |
| ***Generalised Epilepsy*** | |
| F250.00 | Generalised non-convulsive epilepsy |
| F250100 | Pykno-epilepsy |
| F250400 | Juvenile absence epilepsy |
| F250y00 | Other specified generalised non-convulsive epilepsy |
| F250z00 | Generalised non-convulsive epilepsy NOS |
| F251.00 | Generalised convulsive epilepsy |
| F251y00 | Other specified generalised convulsive epilepsy |
| F251z00 | Generalised convulsive epilepsy NOS |
| F132100 | Progressive myoclonic epilepsy |
| F132111 | Unverricht - Lundborg disease |
| F25A.00 | Juvenile myoclonic epilepsy |
| Fyu5000 | [X]Other generalized epilepsy and epileptic syndromes |
| ***Unclassified Epilepsy*** | |
| 1O30.00 | Epilepsy confirmed |
| 667B.00 | Nocturnal epilepsy |
| F250000 | Petit mal (minor) epilepsy |
| F251000 | Grand mal (major) epilepsy |
| F25..00 | Epilepsy |
| F251011 | Tonic-clonic epilepsy |
| F251500 | Tonic-clonic epilepsy |
| F255100 | Sensory induced epilepsy |
| F255300 | Visceral reflex epilepsy |
| F255400 | Visual reflex epilepsy |
| F255012 | Motor epilepsy |
| F25B.00 | Alcohol-induced epilepsy |
| F25C.00 | Drug-induced epilepsy |
| F25D.00 | Menstrual epilepsy |
| F25E.00 | Stress-induced epilepsy |
| F25F.00 | Photosensitive epilepsy |
| F25y.00 | Other forms of epilepsy |
| F25yz00 | Other forms of epilepsy NOS |
| F25z.00 | Epilepsy NOS |
| Fyu5100 | [X]Other epilepsy |

*Supplementary Figure 3: Algorithm for the Assessment of Clinical Investigations*

**PRIMARY CARE Dataset**

**EMERGENCY Datasets**

**Review Investigation Codes:**

***MRI***, ***CT*** or ***EEG*** code listed

**Non-Specific Codes:**

*Codes not specifying anatomical site must correlate with a known episode of seizure occurrence*

**INVESTIGATION PERFORMED**

**Review Investigation Codes:**

***MRI*** or ***CT*** code listed

**Emergency Attendance**

*Consistent with an episode of seizure occurrence*

*Supplementary Table 5: Investigation Codes Included in the Assessment of Clinical Investigations*

| **Code** | **Code Description** | |
| --- | --- | --- |
| ***CT Brain*** | | |
| HES: 12 | | Computed Tomography |
| SAIL: 201 | | Computed Tomography |
| READ: 567 | | Computed Tomography |
| READ: Y72JC | | CT Head |
| READ: Y72JD | | CT Brain |
| READ: YAYCE | | CT of Bone Structures and Cavities of the Head |
| READ: YAYCB | | CT Bone Structures of the Head |
| READ: YAMGZ | | CT Brain Normal |
| READ: YAQSS | | CT Brain Abnormal |
| READ: 5674 | | CT Skull |
| ***MRI Brain*** | | |
| HES: 11 | | Magnetic Resonance Imaging |
| SAIL: 202 | | Magnetic Resonance Imaging |
| READ: 569 | | Magnetic Resonance: (Imaging) or (Study) |
| READ: Y7212 | | MRI of Head |
| READ: Y7213 | | MRI of Brain |
| READ: Y7215 | | MRI of Brain with Functional Imaging |
| READ: YB095 | | MRI Scan Abnormal |
| READ: YB088 | | MRI Scan Normal |
| READ : 5692 | | Nuclear Magnetic Resonance Scan: Normal |
| READ: 5693 | | Nuclear Magnetic Resonance Scan: Abnormal |
| ***EEG*** | | |
| X77i2 | | Scalp EEG |
| X77i8 | | Sleep EEG |
| X77iL | | Video EEG |
| X77iM | | EEG telemetry |
| X77JD | | Asymmetric EEG |
| X77iI | | Ambulatory EEG |
| X77I8 | | EEG observations |
| X77Ir | | Focal EEG pattern |
| X77jK | | Intraoperative EEG |
| X77iL | | EEG video telemetry |
| X77iI | | AEEG - Ambulatory EEG |
| X77Ir | | Localised EEG pattern |
| X77I9 | | Generalised EEG pattern |
| X77iJ | | Continuous processed EEG |
| X77i9 | | Sleep EEG - natural sleep |
| Xa0ej | | Continuous EEG measurements |
| X77i4 | | EEG with photic stimulation |
| 70650 | | EEG - Electroencephalography |
| X77i6 | | EEG with drug administration |
| X77J1 | | Focal episodic EEG abnormality |
| X77Iz | | Focal reduction of EEG activity |
| X77Is | | Focal continuous EEG abnormality |
| X77iA | | Sleep EEG - sleep-deprived patient |
| 31130 | | EEG normal |
| XM18c | | EEG abnormal |
| X77IA | | EEG artefact |
| X77JH | | Generalised EEG frequency asymmetry |
| X77JG | | Generalised EEG amplitude asymmetry |
| X77Ig | | Generalised episodic EEG abnormality |
| X77II | | EEG pattern of uncertain significance |
| X77Ig | | Generalised paroxysmal EEG abnormality |
| X77IQ | | Generalised continuous EEG abnormality |
| X77i7 | | EEG during special activation procedure |
| X77IQ | | Generalised non-paroxysmal EEG abnormality |
| X77i5 | | EEG with over breathing and photic stimulation |
| X77IM | | Subclinical rhythmical EEG discharges in adults |
| X77IM | | SREDA - Subclinical rhythmical EEG discharges in adults |
| 70650 | | Electroencephalography |
| XaPpX | | Electroencephalography NEC |
| 70650 | | EEG - Electroencephalography |
| XM18c | | Electroencephalogram abnormal |
| X77iM | | Electroencephalograph telemetry |

*Supplementary Figure 4: Algorithm for the Identification of Adverse Events*

**Review Emergency Codes**:

Diagnostic code clinically consistent with adverse event recorded in SANAD II and occurring within 90 days of the SANAD II recorded adverse event

**Review READ Codes**:

Diagnostic code clinically consistent with adverse event recorded in SANAD II and occurring within 90 days of the SANAD II recorded adverse event

**Review ICD Codes**:

Diagnostic code clinically consistent with adverse event recorded in SANAD II and occurring within 90 days of the SANAD II recorded adverse event

**Admission**:

Any Admission Method

Any Speciality

**Review ICD Codes**:

Diagnostic code clinically consistent with adverse event recorded in SANAD II and occurring within 90 days of the SANAD II recorded adverse event

OR

OR

OR

**ADVERSE EVENT**

**Review Emergency Codes:**

‘Adverse Event’ code listed

**Review READ Codes:**

‘Adverse Event’ code listed

OR

**Review ICD Codes:**

‘Adverse Event’ code listed

**Admission**:

Any Admission Method

Any Speciality

**Review ICD Codes**:

‘Adverse event’ code listed in any diagnostic position

*Supplementary Table 6: Diagnostic Codes Indicating ‘Adverse Events’*

| **Code** | **Code Description** | |
| --- | --- | --- |
| ***ICD 10 Codes*** | | |
| T88 | | Other complications of surgical and medical care, not elsewhere classified |
| T88.6 | | Anaphylactic reaction due to adverse effect of correct drug or medicament properly administered |
| T88.7 | | Unspecified adverse effect of drug or medicament |
| T88.8 | | Other specified complications of surgical and medical care, not elsewhere classified |
| T88.9 | | Complication of surgical and medical care, unspecified |
| T42.0 | | Poisoning by, adverse effect of and underdosing of hydantoin derivatives |
| T42.1 | | Poisoning by, adverse effect of and underdosing of iminostilbenes |
| T42.2 | | Poisoning by, adverse effect of and underdosing of succinimides and oxazolidinediones |
| T42.3 | | Poisoning by, adverse effect of and underdosing of barbiturates |
| T42.4 | | Poisoning by, adverse effect of and underdosing of benzodiazepines |
| T42.5 | | Poisoning by, adverse effect of and underdosing of mixed antiepileptics |
| T42.6 | | Poisoning by, adverse effect of and underdosing of other antiepileptic and sedative-hypnotic drugs |
| T42.7 | | Poisoning by, adverse effect of and underdosing of unspecified antiepileptic and sedative-hypnotic drugs |
| ***Emergency Code*** | | |
| 32 | | Allergy (anaphylaxis) |
| ***READ Codes*** | | |
| SN5 | | Adverse effects NEC |
| SN5z | | Adverse effects NOS |
| TJ632 | | Adverse reaction to carbamazepine |
| TJ63 | | Adverse reaction to other anticonvulsant |
| TJ61 | | Adverse reaction to hydantoin derivative |
| TJHyz | | Adverse reaction to other drug or medicine NOS |
| TJHz. | | Adverse reaction to drug or medicinal substance NOS |
| TJ6 | | Adverse reaction to anticonvulsants and anti-parkinsonism drugs |
| TJ6z | | Adverse reaction to anticonvulsant and antiparkinsonism drugs NOS |
| Xa5Jh | | Lamotrigine adverse reaction |
| TJ632 | | Adverse reaction to carbamazepine |
| TJ633 | | Adverse reaction to sodium valproate |
| TJ610 | | Adverse reaction to phenytoin |
| TJ70 | | Adverse reaction to barbiturate |
| TJ94 | | Adverse reaction to benzodiazepine-based tranquilliser |

*Supplementary Figure 5: The Identification of the Date of First Seizure in Routine Datasets*

**98 Participants**

**36 Participants**

*SANAD II: First seizure recorded prior to 2013*

**62 Participants**

**11 Participants**

*Routine: No evidence of seizure or relevant attendances with missing or discrepant diagnostic codes*

**51 Participants**

**12 Participants**

*Routine: Relevant attendances, missing diagnostic code:*

- *A&E / EDDS: 12*
- *APC / PEDW: 0*

**16 Participants**

*Routine: Relevant attendances, inadequate or discrepant diagnostic code not meeting the criteria for seizure occurrence:*

- *A&E / EDDS: 16*
- *APC / PEDW: 5*

**23 Participants**

*Relevant attendances for dates of first seizure identified:*

- *HES A&E: 12 SAIL EDDS: 8*
- *HES APC: 10 SAIL PEDW: 5*

*SAIL GP: 6*

*Attendances recorded in 1 dataset (7 participants), 2 datasets (14 participants) and 3 datasets (2 participants)*

Supplementary *Figure 6: The Difference in Days Between the Date of First Seizure*


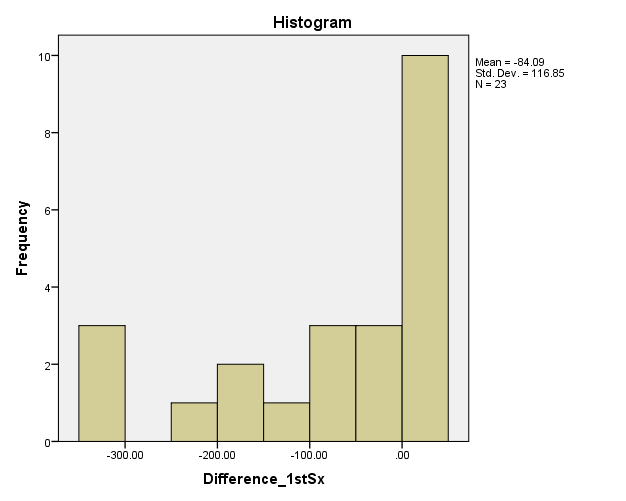


*Supplementary Figure 7: Date of First Seizure: Bland Altman Plot*

| *Mean* | -84.09 |
| --- | --- |
| *Upper 95% Confidence Limit of Agreement* | 144.94 |
| *Lower 95% Confidence Limit of Agreement* | -313.12 |


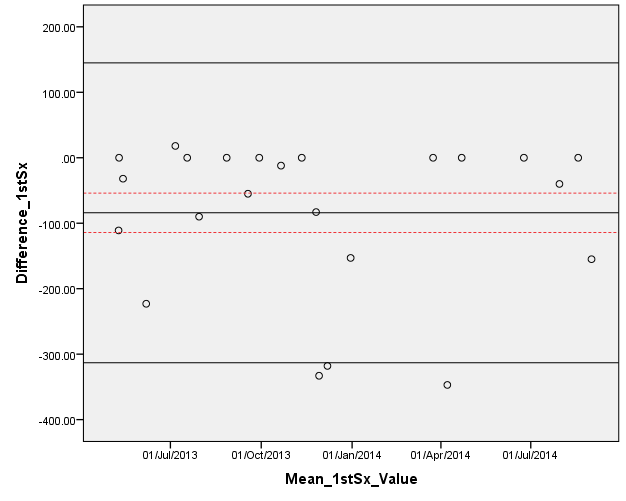


*Supplementary Figure 8: The Identification of the Date of First Tonic-Clonic Seizure in Routine Datasets*

**98 Participants**

**36 Participants**

*SANAD II: First seizure recorded prior to 2013*

**62 Participants**

**11 Participants**

*Routine: No evidence of seizure or relevant attendances with missing or discrepant diagnostic codes*

**51 Participants**

**12 Participants**

*Routine: Relevant attendances, missing diagnostic code:*

- *A&E / EDDS: 12*
- *APC / PEDW: 0*

**16 Participants**

*Routine: Relevant attendances, inadequate or discrepant diagnostic code not meeting the criteria for seizure occurrence:*

- *A&E / EDDS: 16*
- *APC / PEDW: 5*

**23 Participants**

**1 Participant**

*Routine: Single seizure occurrence, no evidence of diagnostic code consistent with tonic-clonic seizure*

**22 Participants**

*Relevant attendances for dates of first tonic-clonic seizure identified:*

- *HES A&E: 12 SAIL EDDS: 8*
- *HES APC: 10 SAIL PEDW: 5*

*SAIL GP: 5*

*Attendances recorded in 1 dataset (6 participants), 2 datasets (14 participants) and 3 datasets (2 participants)*

Supplementary *Figure 9: The Difference in Days Between the Date of First Tonic-Clonic Seizure*


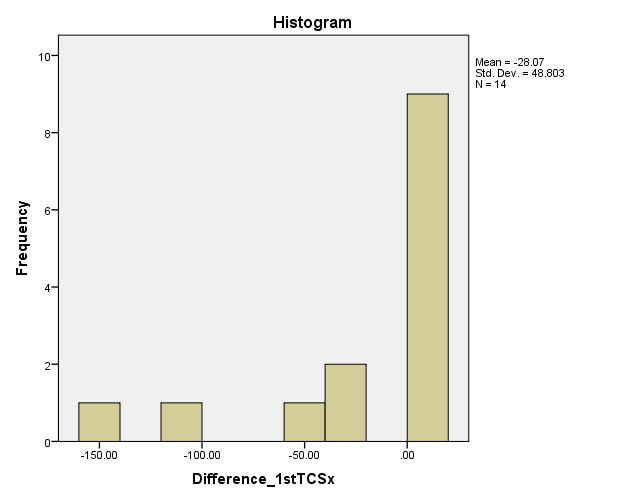


*Supplementary Figure 10: Date of First Tonic-Clonic Seizure: Bland Altman Plot*

| *Mean* | -28.07 |
| --- | --- |
| *Upper 95% Confidence Limit of Agreement* | 67.58 |
| *Lower 95% Confidence Limit of Agreement* | -123.72 |


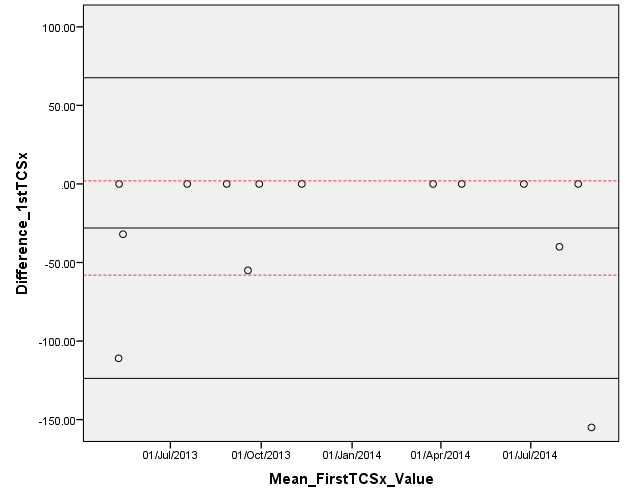


*Supplementary Figure 11: The Identification of the Date of Baseline Diagnosis in Routine Datasets*

**98 Participants**

**20 Participants**

*SANAD II: First seizure recorded prior to 2013*

*Routine: No evidence of diagnosis*

**78 Participants**

**10 Participants**

*Routine: No evidence of diagnostic code, seizure or relevant attendance with missing or discrepant diagnostic codes*

**8 Participants**

*Routine: Single seizure occurrence*

**4 Participants**

*Routine: Single seizure occurrence and relevant single attendance, missing diagnostic code*

**4 Participants**

*Routine: Single seizure occurrence and relevant single attendance, inadequate diagnostic code not meeting the criteria for seizure occurrence*

**2 Participants**

*Routine: Relevant single attendance, missing diagnostic code*

**1 Participant**

*Routine: Two relevant attendances, missing diagnostic code*

**8 Participants**

*Routine: Relevant single attendance, discrepant diagnostic code not meeting the criteria for seizure occurrence*

**4 Participants**

*Routine: Two relevant attendances, one missing diagnostic code, one discrepant diagnostic code not meeting the criteria for seizure occurrence*

**68 Participants**

**37 Participants**

**9 Participants**

*Routine: Evidence of two episodes of seizure*

**28 Participants**

*Routine: Evidence of diagnostic code*

Supplementary *Figure 12: The Difference in Days Between the Dates of Baseline Diagnosis of Epilepsy*


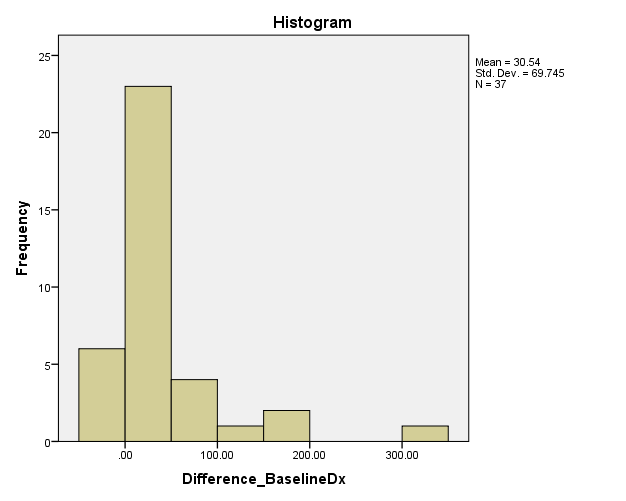


*Supplementary Figure 13: Baseline Diagnosis of Epilepsy: Bland Altman Plot*

| *Mean* | 30.54 |
| --- | --- |
| *Upper 95% Confidence Limit of Agreement* | 167.25 |
| *Lower 95% Confidence Limit of Agreement* | -106.17 |


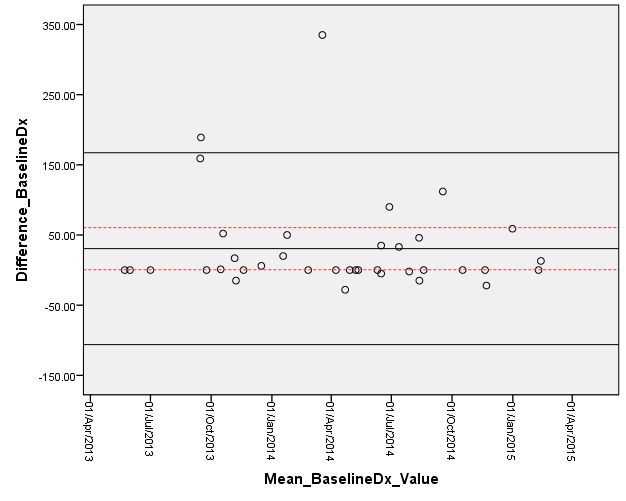


*Supplementary Figure 13: The Identification of the Date of All-Time Diagnosis in Routine Datasets*

**8 Participants**

*Routine: Single seizure occurrence*

**1 Participant**

*Routine: Single seizure occurrence and relevant single attendance, missing diagnostic code*

**2 Participants**

*Routine: Single seizure occurrence and two relevant attendances, missing diagnostic codes*

**2 Participants**

*Routine: Single seizure occurrence and relevant single attendance, inadequate diagnostic code not meeting the criteria for seizure occurrence*

**2 Participants**

*Routine: Single seizure occurrence, two relevant attendances, one missing diagnostic code, one discrepant diagnostic code not meeting the criteria for seizure occurrence*

**1 Participant**

*Routine: Single seizure occurrence, three relevant attendances, inadequate diagnostic codes not meeting the criteria for seizure occurrence*

**1 Participant**

*Routine: Single seizure occurrence, three relevant attendances, two missing diagnostic codes, one discrepant diagnostic code not meeting the criteria for seizure occurrence*

**1 Participant**

*Routine: Relevant single attendance, missing diagnostic code*

**7 Participants**

*Routine: Relevant single attendance, discrepant diagnostic code not meeting the criteria for seizure occurrence*

**3 Participants**

*Routine: Two relevant attendances, one missing diagnostic code, one discrepant diagnostic code not meeting the criteria for seizure occurrence*

**98 Participants**

**17 Participants**

*SANAD II: First seizure recorded prior to 2013*

*Routine: No evidence of diagnosis*

**81 Participants**

**6 Participants**

*Routine: No evidence of diagnostic code, seizure or relevant attendance with missing or discrepant diagnostic codes*

**75 Participants**

**47 Participants**

**11 Participants**

*Routine: Evidence of two episodes of seizure*

**36 Participants**

*Routine: Evidence of diagnostic code*

Supplementary *Figure 14: The Difference in Days Between the Dates of All-Time Diagnosis of Epilepsy*


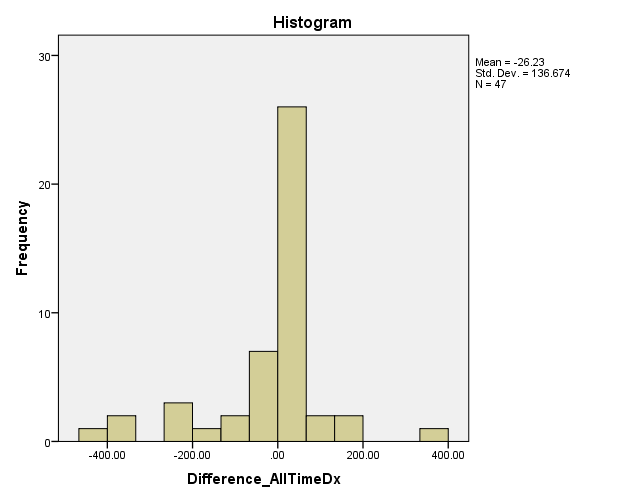


*Supplementary Figure 15: All-Time Diagnosis of Epilepsy: Bland Altman Plot*

| *Mean* | -26.23 |
| --- | --- |
| *Upper 95% Confidence Limit of Agreement* | 241.64 |
| *Lower 95% Confidence Limit of Agreement* | -294.10 |


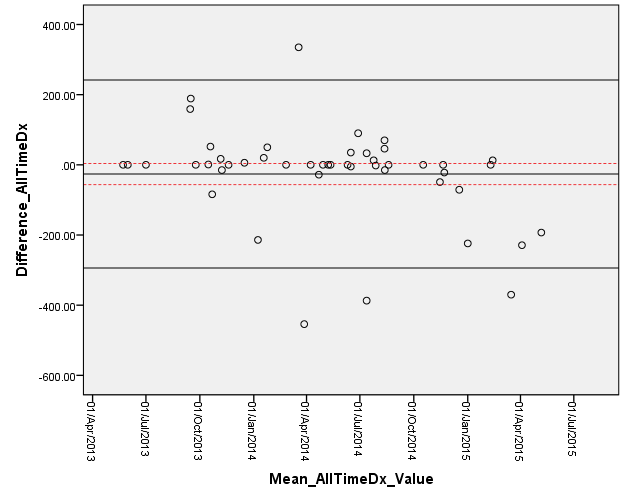


*Supplementary Table 7: Baseline Classification: Cross-Tabulation*

| **RCT Data** |  | **Routine Data** | | | |
| --- | --- | --- | --- | --- | --- |
|  |  | **Focal** | **Generalised** | **Unclassified** | **Total** |
|  | **Focal** | 17 (46.0%) | 1 (2.7%) | 15 (40.6%) | 33 (89.2%) |
|  | **Generalised** | 0 | 1 (2.7%) | 2 (5.4%) | 3 (8.1%) |
|  | **Unclassified** | 0 | 0 | 1 (2.7%) | 1 (2.7%) |
|  | **Total** | 17 (46.0%) | 2 (5.4%) | 18 (48.6%) | 37 |

*Supplementary Table 8: All-Time Classification: Cross-Tabulation*

| **RCT Data** |  | **Routine Data** | | | |
| --- | --- | --- | --- | --- | --- |
|  |  | **Focal** | **Generalised** | **Unclassified** | **Total** |
|  | **Focal** | 22 (46.8%) | 2 (4.3%) | 19 (40.4%) | 43 (91.5%) |
|  | **Generalised** | 0 | 1 (2.1%) | 2 (4.3%) | 3 (6.4%) |
|  | **Unclassified** | 0 | 0 | 1 (2.1%) | 1 (2.1%) |
|  | **Total** | 22 (46.8%) | 3 (6.4%) | 22 (46.8%) | 47 |

*Supplementary Table 9: MRI: Cross-Tabulation*

| **RCT Data** | **Routine Data** | | | |
| --- | --- | --- | --- | --- |
|  |  | **MRI Performed** | **MRI Not Performed** | **Total** |
|  | **MRI Performed** | 5 (5.5%) | 67 (73.6%) | 72 (79.1%) |
|  | **MRI Not Performed** | 2 (2.2%) | 17 (18.7%) | 19 (20.9%) |
|  | **Total** | 7 (7.7%) | 84 (92.3%) | 91 |

*Supplementary Table 10: CT: Cross-Tabulation*

| **RCT Data** | **Routine Data** | | | |
| --- | --- | --- | --- | --- |
|  |  | **CT**  **Performed** | **CT Not Performed** | **Total** |
|  | **CT Performed** | 18 (19.8%) | 15 (16.5%) | 33 (36.3%) |
|  | **CT Not Performed** | 9 (9.9%) | 49 (53.8%) | 58 (63.7%) |
|  | **Total** | 27 (29.7%) | 64 (70.3%) | 91 |

*Supplementary Table 11: EEG: Cross-Tabulation*

| **RCT Data** | **Routine Data** | | | |
| --- | --- | --- | --- | --- |
|  |  | **EEG**  **Performed** | **EEG Not Performed** | **Total** |
|  | **EEG Performed** | 7 (31.8%) | 11 (50%) | 18 (81.8%) |
|  | **EEG Not Performed** | 0 | 4 (18.2%) | 4 (18.2%) |
|  | **Total** | 7 (31.8%) | 15 (68.2%) | 22 |

*Supplementary Figure 16: The Identification of the Date of First Follow-Up Seizure in Routine Datasets*

**98 Participants**

**70 Participants**

*Routine: No evidence of seizure or relevant attendances with missing or discrepant diagnostic codes*

**28 Participants**

**3 Participants**

*Routine: Single relevant attendance, missing diagnostic code:*

- *A&E / EDDS: 3*
- *APC / PEDW: 0*

**3 Participants**

*Routine: Single relevant attendance, diagnostic code not meeting the criteria for seizure occurrence:*

- *A&E / EDDS: 3*
- *APC / PEDW: 0*

**22 Participants**

*Relevant attendances for dates of seizure identified:*

- *HES A&E: 11 SAIL EDDS: 1*
- *HES APC: 9 SAIL PEDW: 3*

*SAIL GP: 4*

*Attendances recorded in 1 dataset (16 participants) and 2 datasets (6 participants)*

**22 Participants**

Supplementary *Figure 17: The Difference in Days Between the Date of First Follow-Up Seizure*


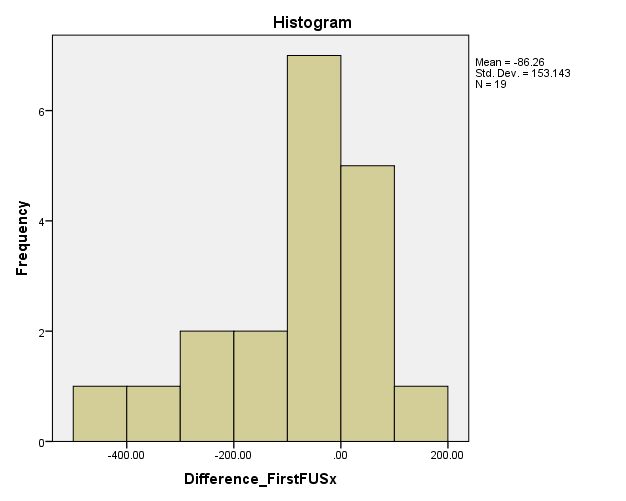


*Supplementary Figure 18: Date of First Follow-Up Seizure: Bland Altman Plot*

| *Mean* | -86.26 |
| --- | --- |
| *Upper 95% Confidence Limit of Agreement* | 213.89 |
| *Lower 95% Confidence Limit of Agreement* | -386.41 |


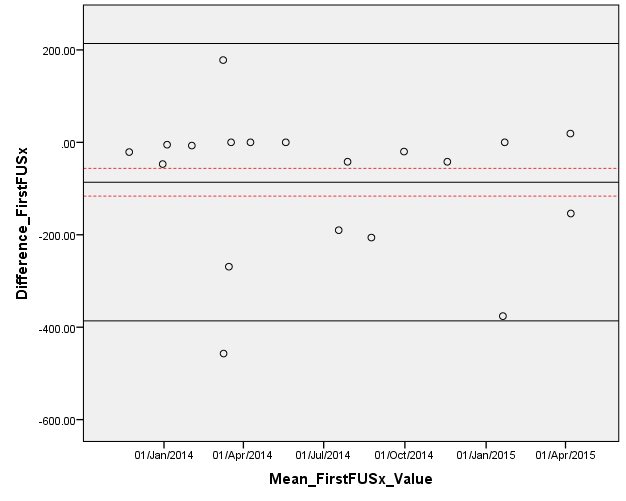


*Supplementary Table 12: The Time to First Follow-Up Seizure: Descriptive Statistics*

|  | **Total:**  **Included Patients** | **Total:**  **Experiencing First Follow-Up Seizure** | **Total:**  **Censored (%)** | **Mean**  **(95% CI)** | **Median**  **(95% CI)** |
| --- | --- | --- | --- | --- | --- |
| **RCT Data** | 98 | 61 | 37 (37.8%) | 325 (258-393) | 111 (38-184) |
| **Routine Data** | 98 | 22 | 76 (77.6%) | 751 (680-822) | N/A |

*Supplementary Figure 19: Kaplan Meier Curve: The Time to First Follow-Up Seizure*


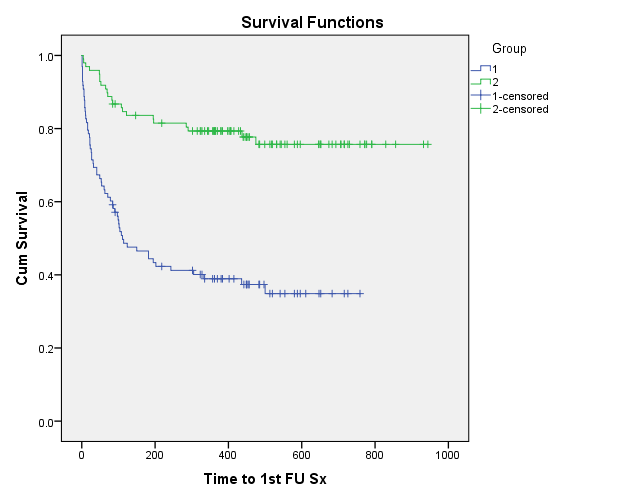


**1 = SANAD II Dataset**

**2 = Routine Dataset**

*Supplementary Figure 19: The Identification of the Date of First Follow-Up Tonic-Clonic Seizure in Routine Datasets*

**98 Participants**

**70 Participants**

*Routine: No evidence of seizure or relevant attendances with missing or discrepant diagnostic codes*

**28 Participants**

**3 Participants**

*Routine: Single relevant attendance, missing diagnostic code:*

- *A&E / EDDS: 3*
- *APC / PEDW: 0*

**3 Participants**

*Routine: Single relevant attendance, diagnostic code not meeting the criteria for seizure occurrence:*

- *A&E / EDDS: 3*
- *APC / PEDW: 0*

**20 Participants**

*Relevant attendances for dates of seizure identified:*

- *HES A&E: 10 SAIL EDDS: 1*
- *HES APC: 9 SAIL PEDW: 3*

*SAIL GP: 2*

*Attendances recorded in 1 dataset (15 participants) and 2 datasets (5 participants)*

**2 Participants**

*Routine: Single follow-up seizure occurrence, no evidence of diagnostic codes consistent with tonic-clonic seizure occurrence*

**20 Participants**

**22 Participants**

Supplementary *Figure 20: The Difference in Days Between the Date of First Follow-Up Tonic-Clonic Seizure*


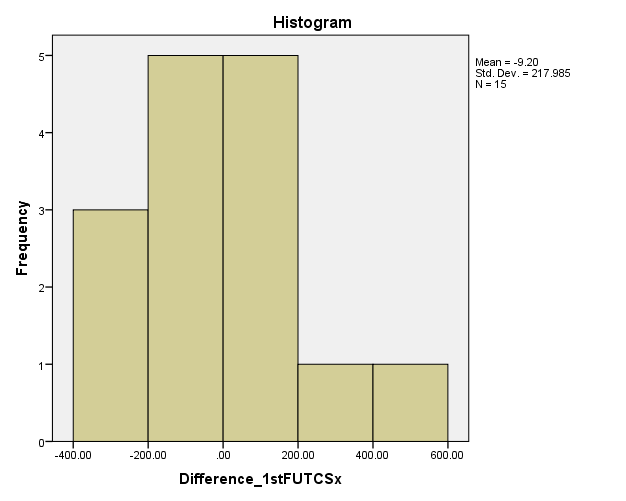


*Supplementary Figure 21: Date of First Follow-Up Tonic-Clonic Seizure: Bland Altman Plot*

| *Mean* | -9.20 |
| --- | --- |
| *Upper 95% Confidence Limit of Agreement* | 418.06 |
| *Lower 95% Confidence Limit of Agreement* | -436.46 |


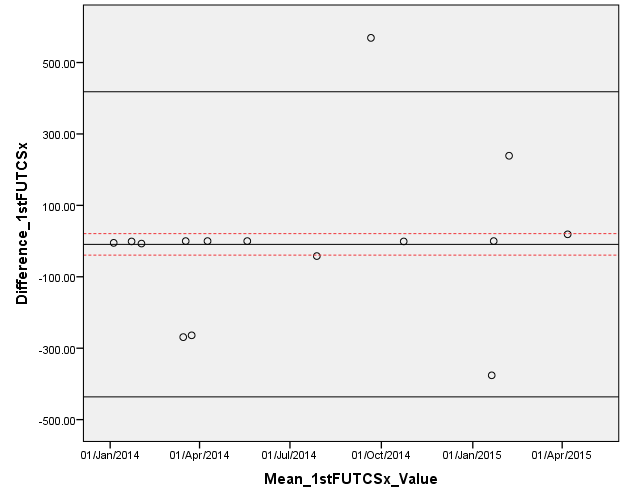


*Supplementary Table 13: The Identification of the Date 12 Month Remission Achieved*

|  | **SANAD II Dataset** | **Routine Datasets** |
| --- | --- | --- |
| **Total Participants** | 98 | 98 |
| **Participants Not Achieving Remission:**  ***Occurrence of Seizures***  ***No Occurrence of Seizures:***   - Insufficient SANAD II Follow-Up   (<12 months SANAD II follow-up)   - Insufficient Routine Data   (‘Remission’ occurring >31/12/15) | 39 (39.8%)  11 (11.2%)  2 (2.0%) | 3 (3.1%)  14 (14.3%)  7 (7.1%) |
| **Participants Achieving Remission** | 46 (46.9%) | 74 (75.5%) |

Supplementary *Figure 22: The Difference in Days Between the Date 12 Month Remission Achieved*


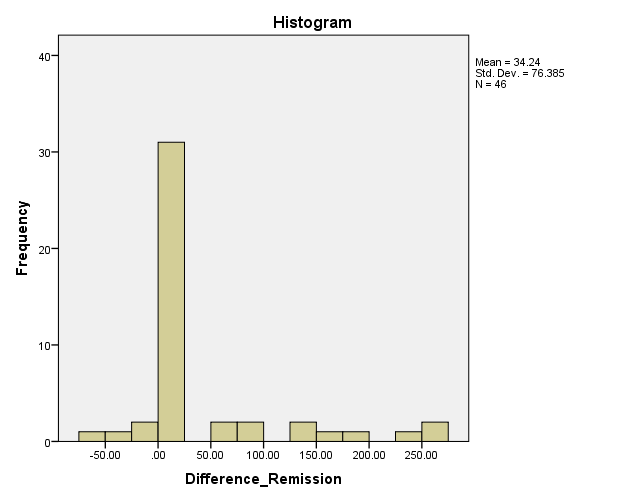


*Supplementary Figure 23: The Date 12 Month Remission Achieved: Bland Altman Plot*

| *Mean* | 34.24 |
| --- | --- |
| *Upper 95% Confidence Limit of Agreement* | 183.96 |
| *Lower 95% Confidence Limit of Agreement* | -115.48 |


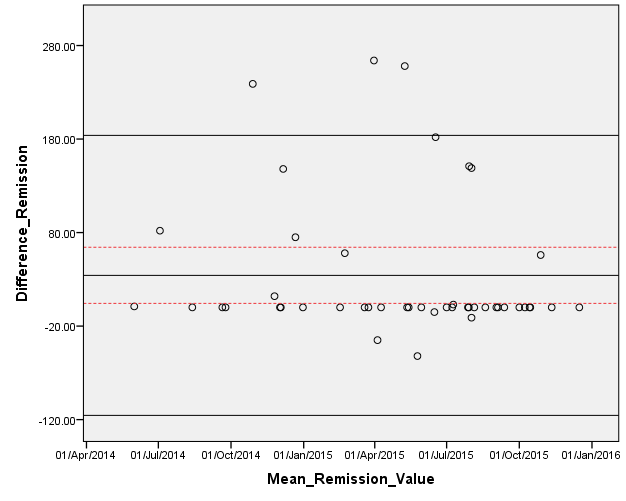


*Supplementary Table 14: The Time to 12 Month Remission: Descriptive Statistics*

|  | **Total:**  **Included Patients** | **Total:**  **Achieving 12 Month Remission** | **Total:**  **Censored (%)** | **Mean**  **(95% CI)** | **Median**  **(95% CI)** |
| --- | --- | --- | --- | --- | --- |
| **RCT Data** | 98 | 46 | 52 (26.5%) | 567 (515-618) | 447 |
| **Routine Data** | 98 | 74 | 24 (12.2%) | 393 (375-410) | 365 |

*Supplementary Figure 24: Kaplan Meier Curve: The Time to 12 Month Remission*


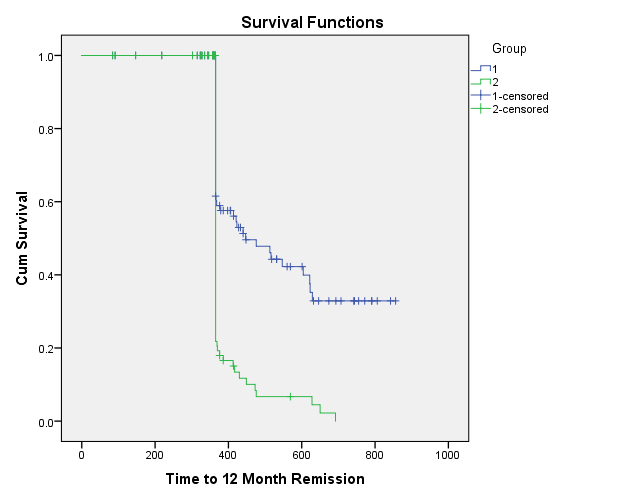


**1 = SANAD II Dataset**

**2 = Routine Dataset**

*Supplementary Figure 25: The Difference in Date of AED First Prescription*


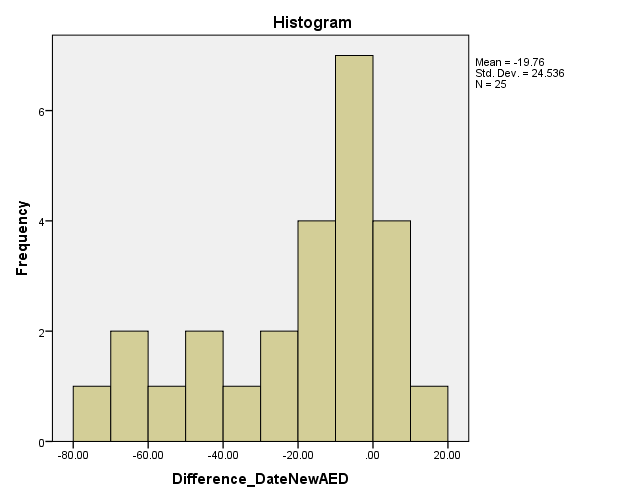


*Supplementary Figure 26: Dates of AED First Prescription: Bland Altman Plot*

| *Mean* | -19.76 |
| --- | --- |
| *Upper 95% Confidence Limit of Agreement* | 28.34 |
| *Lower 95% Confidence Limit of Agreement* | -67.86 |


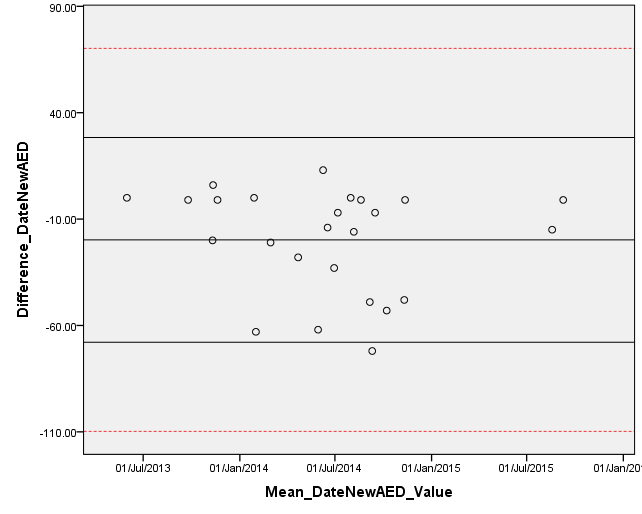


*Supplementary Figure 27: The Difference in Days Between the Date of Baseline Assessment*


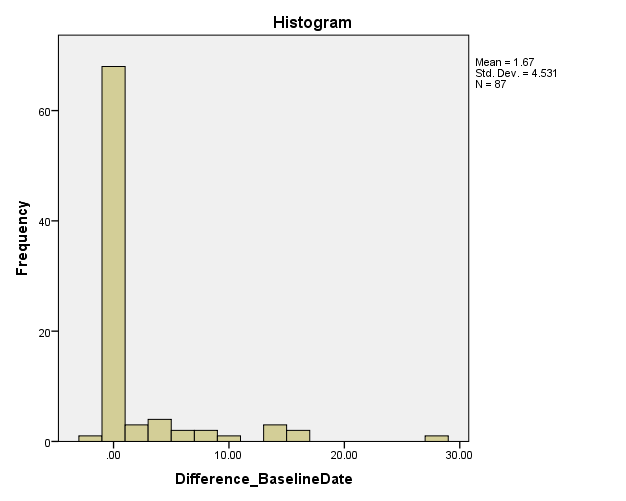


*Supplementary Figure 28: Date of Baseline Assessment: Bland Altman Plot*

| *Mean* | 1.67 |
| --- | --- |
| *Upper 95% Confidence Limit of Agreement* | 10.55 |
| *Lower 95% Confidence Limit of Agreement* | -7.21 |


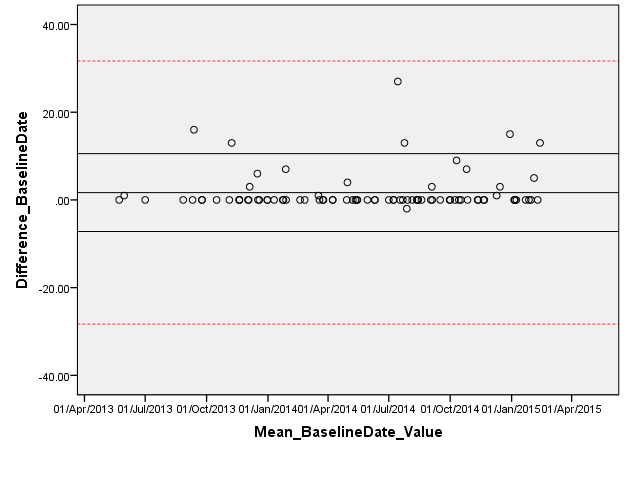


*Supplementary Figure 29: The Difference in Days Between the Date of Follow-Up Assessments*


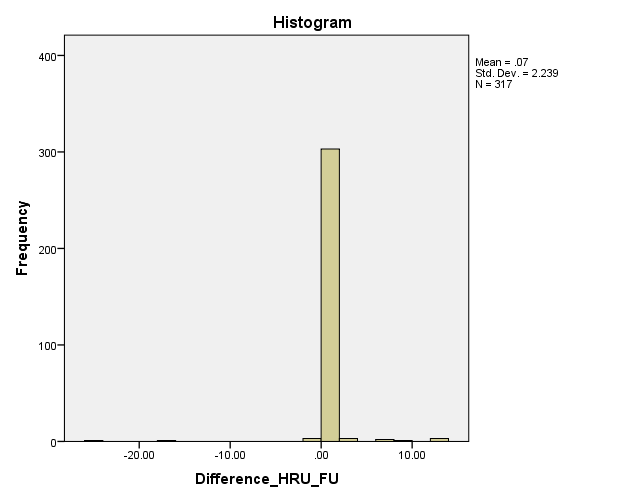


*Supplementary Figure 30: Date of Follow-Up Assessments: Bland Altman Plot*

| *Mean* | 0.07 |
| --- | --- |
| *Upper 95% Confidence Limit of Agreement* | 4.47 |
| *Lower 95% Confidence Limit of Agreement* | -4.33 |


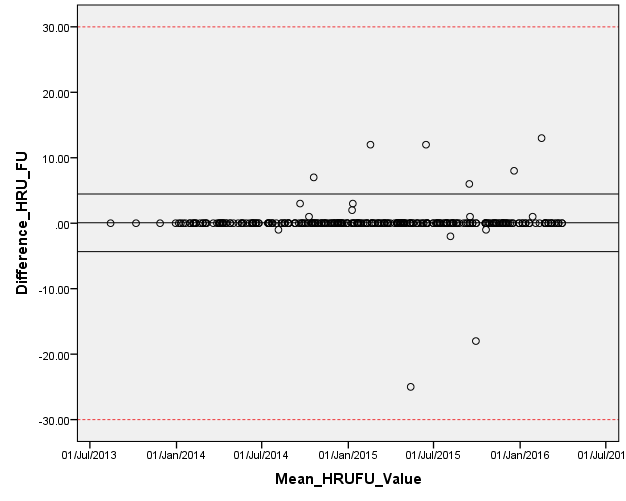


*Supplementary Figure 31: The Difference in Number of Emergency Department Attendances*


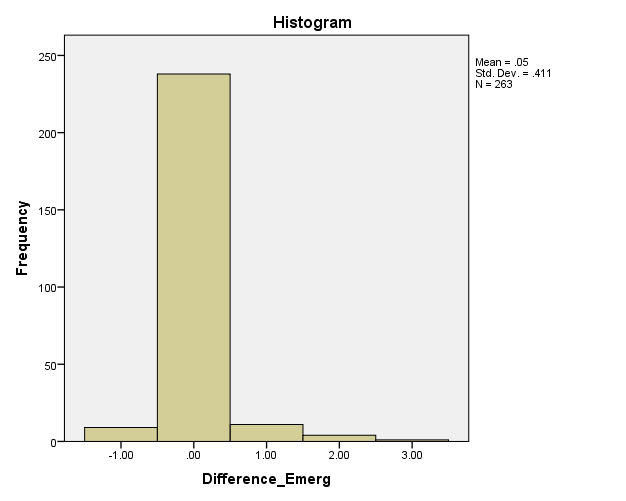


*Supplementary Figure 32: Number of Emergency Department Attendances: Bland Altman Plot*

| *Mean* | 0.05 |
| --- | --- |
| *Upper 95% Confidence Limit of Agreement* | 0.834 |
| *Lower 95% Confidence Limit of Agreement* | -0.734 |


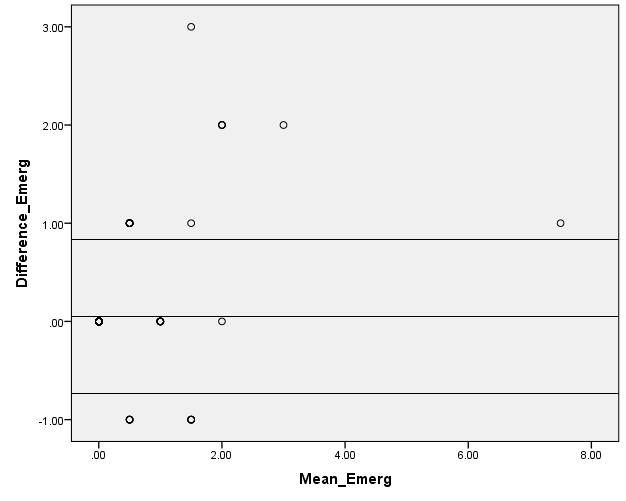


*Supplementary Figure 33: The Difference in Number of Inpatient Admissions*


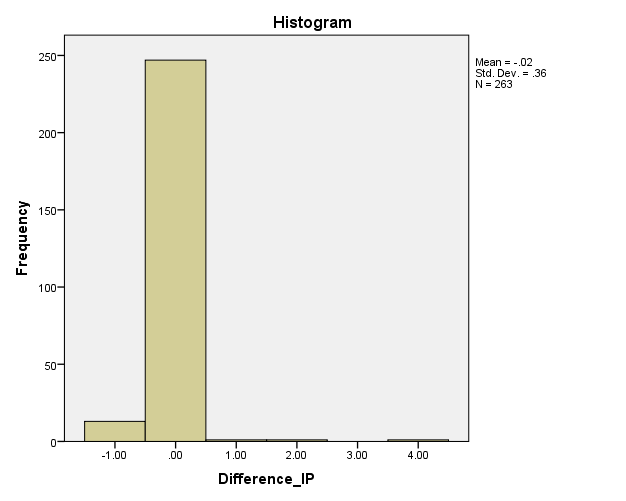


*Supplementary Figure 34: Number of Inpatient Admissions: Bland Altman Plot*

| *Mean* | -0.02 |
| --- | --- |
| *Upper 95% Confidence Limit of Agreement* | 0.68 |
| *Lower 95% Confidence Limit of Agreement* | -0.72 |


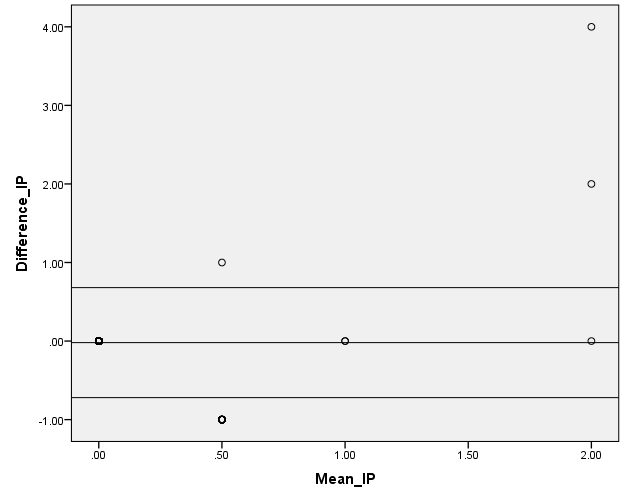

Supplement: Supplementary file 1 — Additional file 1. [file 13063_2021_5294_MOESM1_ESM.docx]
